# Supplementary material for: Hypoxia-induced mobilization of NHE6 to the plasma membrane triggers endosome hyperacidification and chemoresistance
Source: Nat Commun. 2017 Jun 21;8:15884. doi: 10.1038/ncomms15884 (PMC5482059; doi:10.1038/ncomms15884)
Supplement: Supplementary Information [file ncomms15884-s1.pdf]

Type of file: pdf
Title of file for HTML: Supplementary Information
Description: Supplementary Figures and Supplementary Tables

Type of file: pdf
Title of file for HTML: Peer Review File
Description:

Supplementary Table 1: pH measurements in cytosol and endosomal compartments in MDA-MB-231 and HT-1080 cells exposed to 1% or 21% O_2 for 4h.

	**MDA-MB-231**		**HT-1080**	
	**21% O_2**	**1% O_2**	**21% O_2**	**1% O_2**
**Cytosol**	7.03 +/- 0.14	7.36 +/- 0.17	6.79 +/- 0.19	7.22 +/-0.13
**Endosome**	6.57 +/- 0.10	6.17 +/- 0.06	6.08 +/- 0.18	5.47 +/- 0.15
**ΔpH (Cytosol-Endosome)**	0.46	1.19	0.71	1.75
**Δ(ΔpH) (1%0_2-21%0_2)**	**0.73**		**1.03**	

Supplementary Table 2: IC_{50} values of doxorubicin in NHE6 or NHE9 knockdown cells cultured in normoxia or hypoxia.

**Experimental condition**	**IC_{50} DOX (nM)**	**Fold (compared to scrambled 21% O2)**	***p*-value**
scrambled shRNA 21% O_2	178.8 +/- 17.1	**1.0**	-
scrambled shRNA 1% O_2	458.8 +/- 64.7	**2.5**	0.0001
shRNA NHE6 21% O_2	421.4 +/- 27.8	**2.4**	0.0003
shRNA NHE9 21% O_2	201.5 +/- 29.4	**1.1**	ns

Data are presented at the mean (nM) +/- standard deviation. IC_{50}, half maximal inhibitory concentration (n =3 independent experiments with 3 replicates in each experiment). P-values were determined with unpaired *t*-test with Welch's correction.

Supplementary Table 3: IC_{50} values of doxorubicin in HT-1080 overexpressing $NHE6^{527-588}$ peptide.

**IC50 (nM)**	scr	$NHE6^{527-588}$
**21% O2**	117.2 +/- 19.8	136.9 +/- 20.4
**1% O2**	521.1 +/- 28.9	224.1 +/- 22.8
**Fold**	**4.4**	**1.6**
***p* value**	<0.0001	0.0081

Data are presented at the mean (nM) +/- standard deviation. IC_{50}, half maximal inhibitory concentration (n =3 independent experiments with 3 replicates in each experiment). P-values were determined with unpaired *t*-test with Welch's correction.

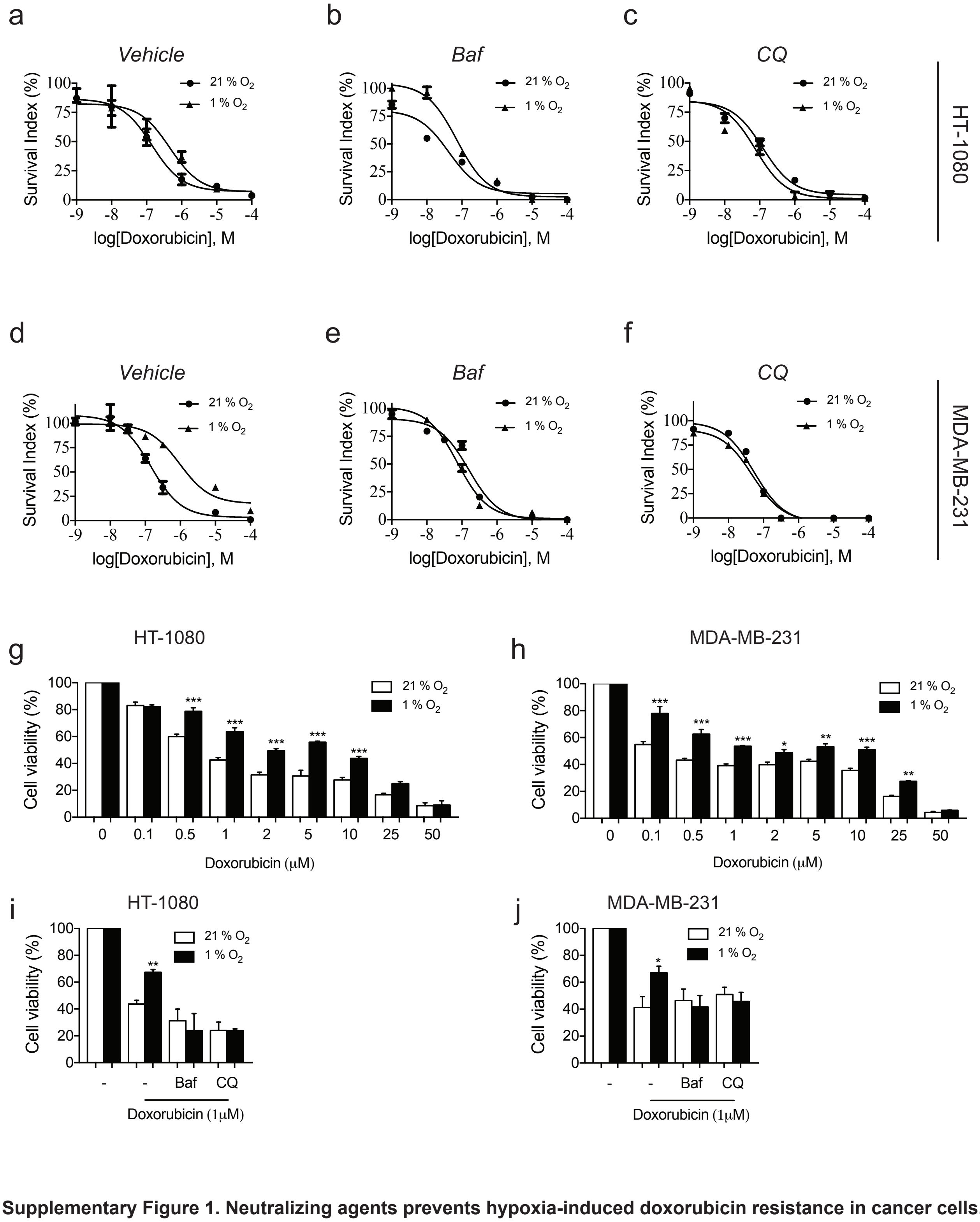

Supplementary Figure 1. Neutralizing agents prevents hypoxia-induced doxorubicin resistance in cancer cells. (a-f) MTT assay dose-response curves for HT-1080 (a-c) and MDA-MB-231 cells (d-f) cultured in normoxia or hypoxia for 72h and in presence or absence of increasing Dox concentrations (n= 3-5 independent experiments. (g-j) Cell viability using the trypan blue exclusion method for HT-1080 (g-i) and MDA-MB-231 cells (h-j) cultured under normoxic or hypoxic conditions in presence or absence of neutralizing agents: Chloroquine (Cq) (10 μM) or Bafilomycin A1 (Baf) (100 nM) (i,j). (n= 3-4 independent experiments). Bars represent the mean ± SEM (* P< 0.05, ** P< 0.01, *** P< 0.001, unpaired Student's t-test).

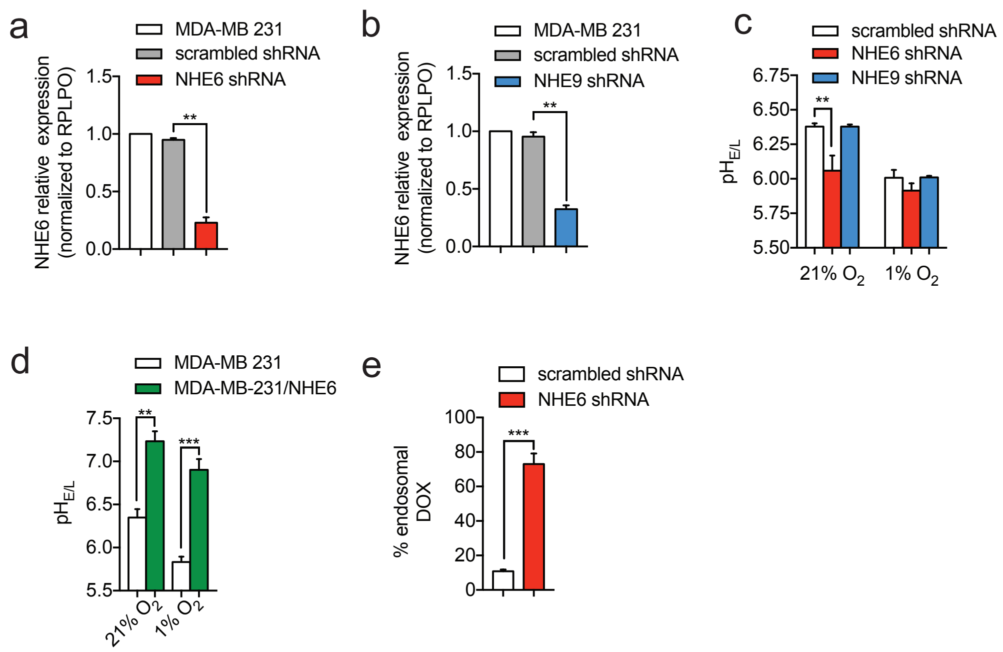

Supplementary Figure 2. NHE6 is a key NHE involved endosome acidification and Dox sequestration.
(a-b) mRNA levels of NHE6 in MDA-MB-231 cells stably transfected with shRNA directed against NHE6 (a),NHE9 (b) or scrambled shRNA. RPLPO was used as control for qPCR (n=3 independent experiments). (c) Endosomal/lysosomal pH in NHE6- or NHE9-knockdown cells cultured under normoxic or hypoxic conditions for 4 h (n =3 independent experiments with > 75 cells/experimental condition). (d) Endosomal/lysosomal pH in NHE6- overexpressing cells cultured under normoxic or hypoxic conditions for 4 h (n =3 independent experiments with > 75 cells/experimental condition). (e) Percentage of Dox fluorescence within DAPI-stained nucleus of NHE6-knockdown cells cultured under normoxic or hypoxic conditions (n =4 independent experiments with > 30 cells/experimental condition). Bars represent the mean ± SEM (** P< 0.01, *** P< 0.001, unpaired Student’s t-test).

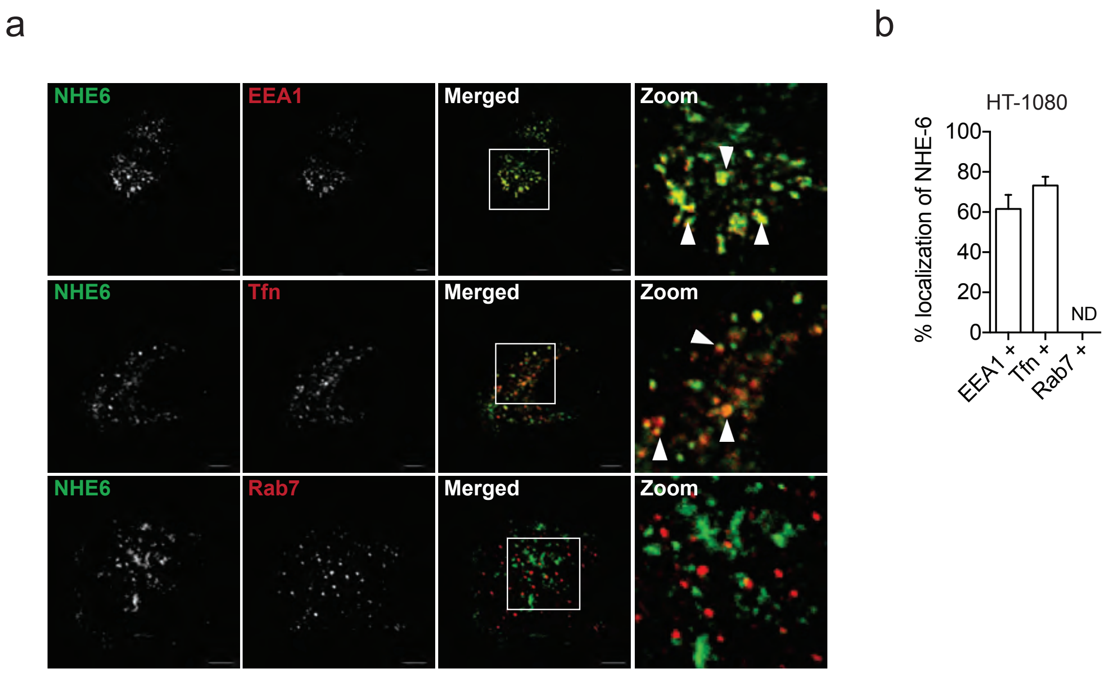

Supplementary Figure 3. NHE6 is predominantly located at early/recycling endosomes in HT-1080 cells. (a) Representative confocal microscopy images of NHE6 localization in HT-1080 cells transfected with NHE6-GFP and labeled with EEA1-directed antibodies (early endosomes), Alexa546-conjugated Tfn (early/recycling endosomes) or Rab7 antibodies (late endosomes) Arrows indicate colocalization of NHE6-GFP with the endosomal markers. Scale bar is 10µm and magnification 60X. (b) Percentage of NHE6-GFP staining in early (EEA+), recycling (Tfn+) and late endosomes (Rab7+) in HT-1080 cells (n = 3 independent experiments with ≥ 75 cells/experimental condition). ND, not detectable.

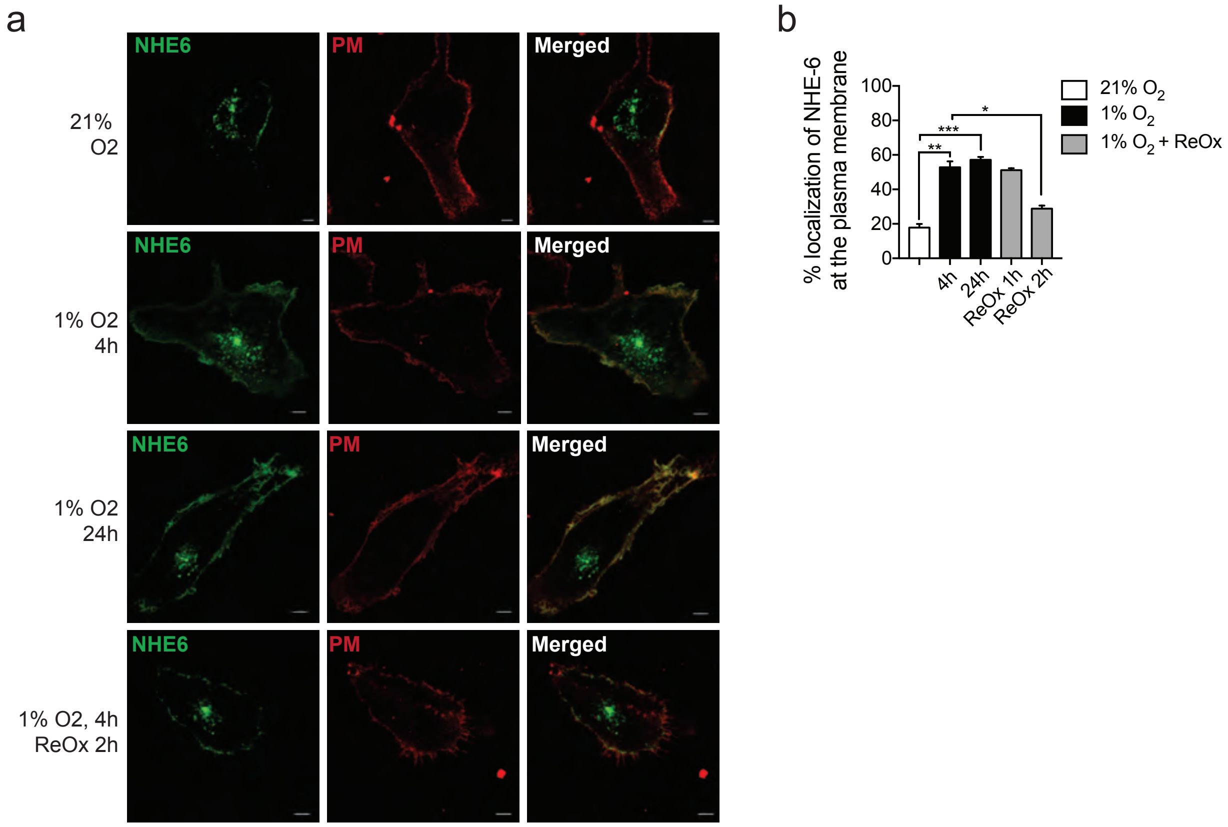

Supplementary Figure 4. Plasma membrane relocalization of NHE6 in hypoxia is a reversible event. (a) HT-1080 cells stably transfected with NHE6-GFP were incubated under normoxic or hypoxic conditions for 4h or 24h. The 4h time point was followed by reoxygenation for 1h or 2h or cells. Representative confocal images of NHE6 localization in HT-1080 cells. Plasma membrane (PM) was stained by cell-surface biotinylation. Scale bar is 5μm and magnification 60X. (b) Percentage of NHE6 at the plasma membrane of HT-1080 cells (n =2-3 independent experiments with > 25 cells/experimental condition). Bars represent the mean ± SEM (** P< 0.01, *** P< 0.001, unpaired Student's t-test).

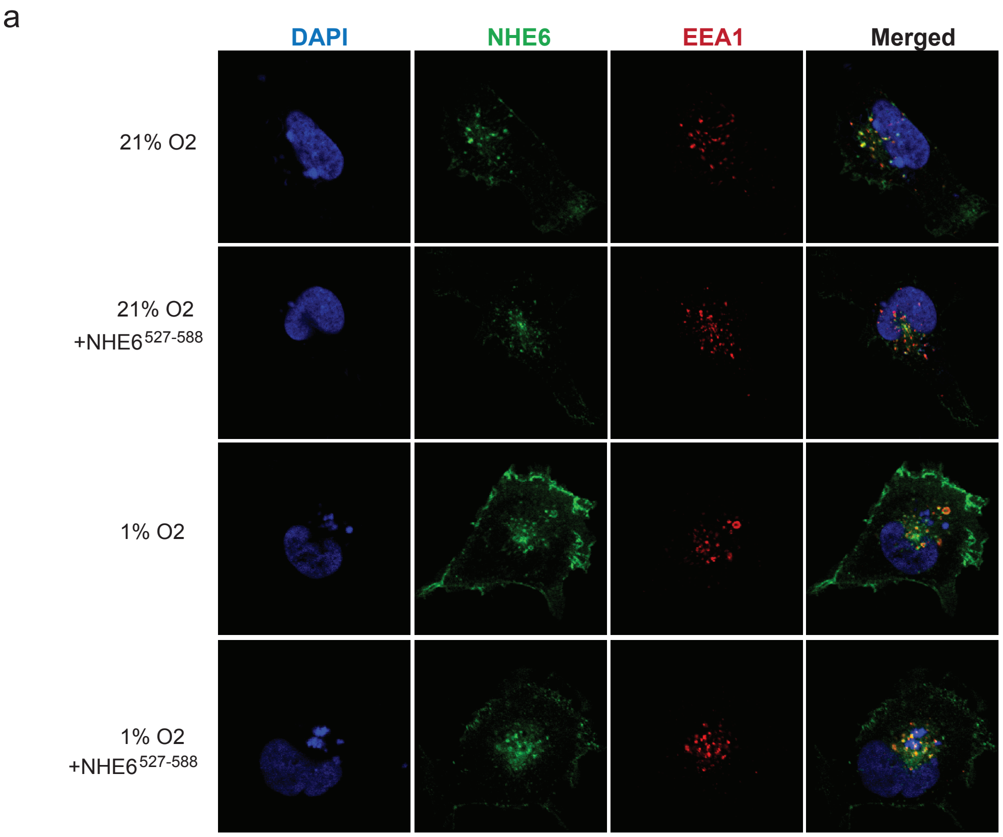

Supplementary Figure 5. The NHE6527-588 peptide blocks NHE6 relocalization in hypoxic cells. Representative confocal microscopy images of NHE6 localization with EEA1 in HT-1080 cells stably transfected with NHE6-GFP and transiently transfected with a plasmid encoding the NHE6527-588 peptide or a scrambled peptide and incubated under 1% O2 or 21% O2 for 4 h. Scale bar is 10μm with magnification 60X.

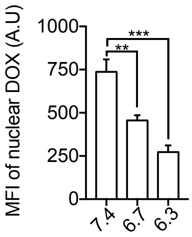

Supplementary Figure 6. Extracellular acidification reduces Dox uptake in HT-1080 cells. Mean fluorescence intensities of nuclear Dox were measured in HT-1080 cells incubated for 4h in the presence of media of different pHs as indicated in the figure. (n = 3 independent experiments with > 20 cells/experimental condition). Bars represent the mean ± SEM (* P<0.05, ** P< 0.01, *** P< 0.001, One-way ANOVA).

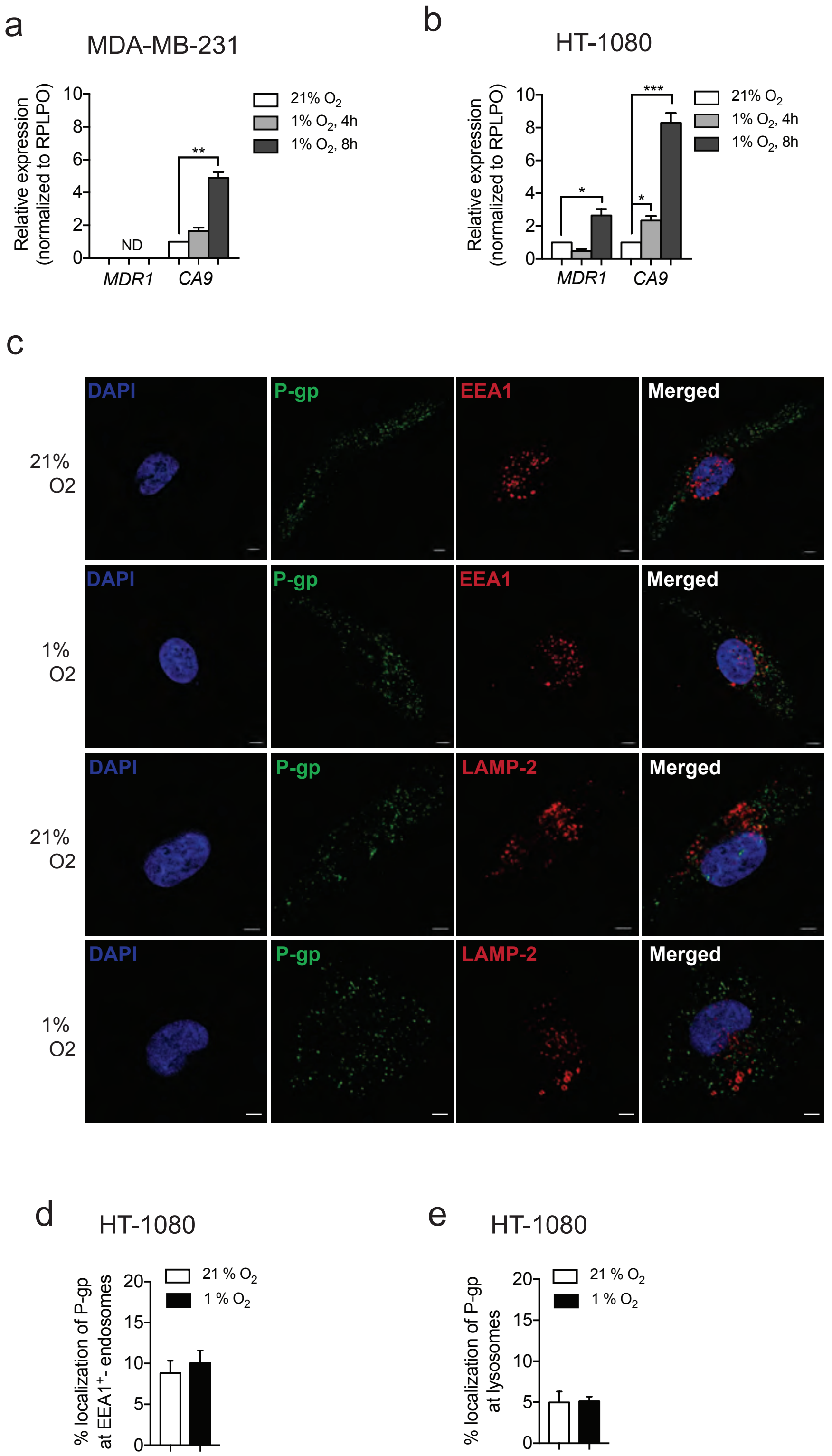

Supplementary Figure 7. Lack of expression of P-glycoprotein is endosomal or lysosomal compartments. (a-b) mRNA levels od MDR1 in MDA-MB-231 (a) and HT-1080 cells (b) cultured in normoxia or hypoxia for 4h or 8h. CA9 mRNA expression was used as a positive control. RPLPO was used as an internal control for qPCR (n=3 independent experiments). (c) Representative confocal microscopy images of P-gp localization in HT-1080 cells labeled with Ab directed against EEA1 (early endosomes) or LAMP2 (lysosomes). Scale bar =10μm and magnification is 60X. (d-e) Percentage of MDR1 staining in early endosomes (EEA+) and lysosomes (LAMP2+) in HT-1080 cells (n = 2 independent experiments with > 20 cells/experimental condition). ND, not detectable. Bars represent the mean ± SEM (* P<0.05, ** P< 0.01, *** P< 0.001, unpaired Student's t-test)

FIGURE 5a

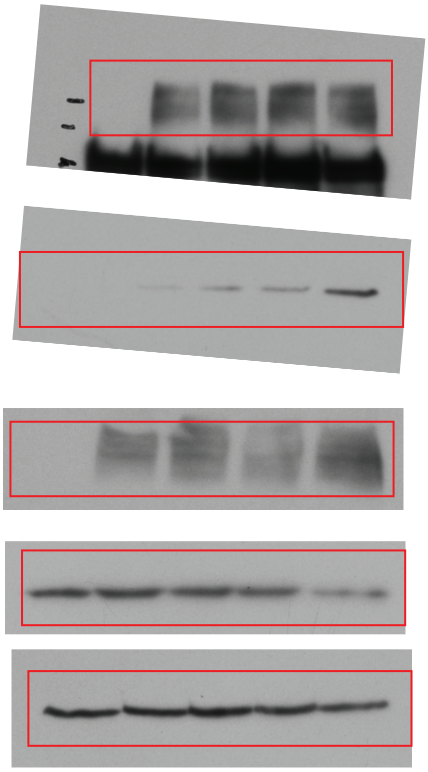

FIGURE 6a

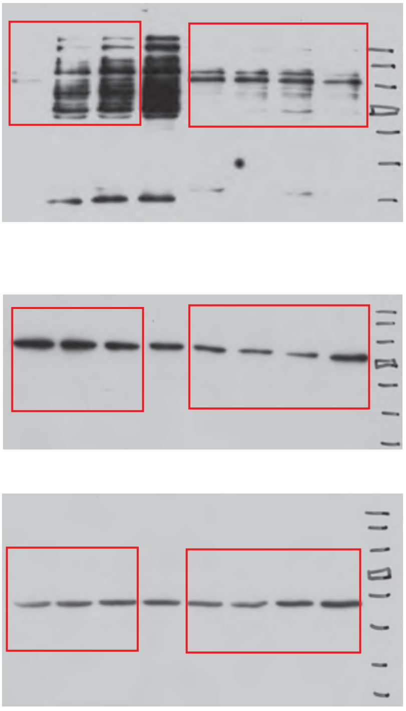

FIGURE 6b

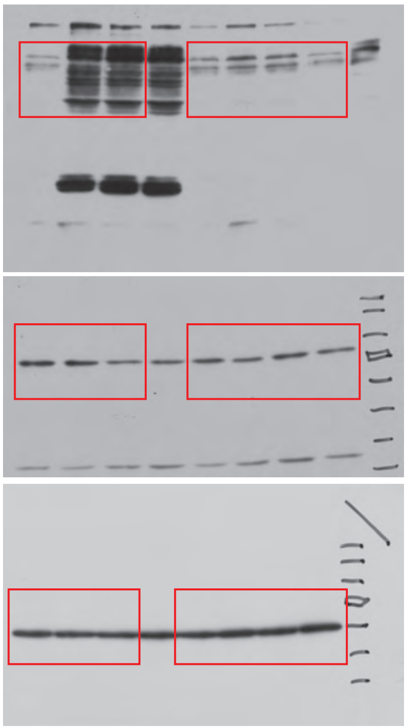

FIGURE 6d

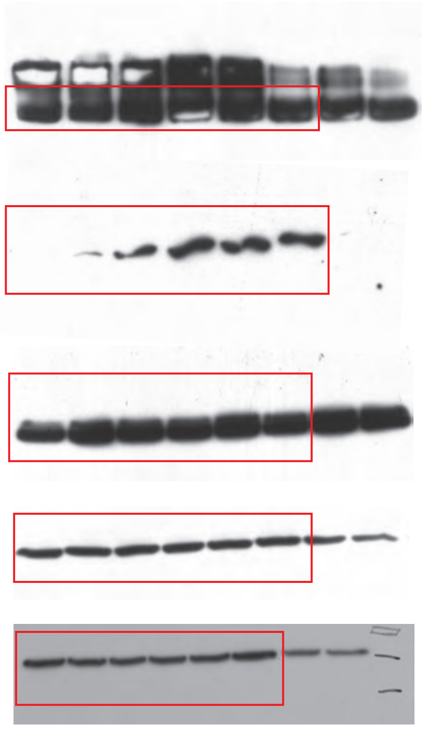

FIGURE 7a

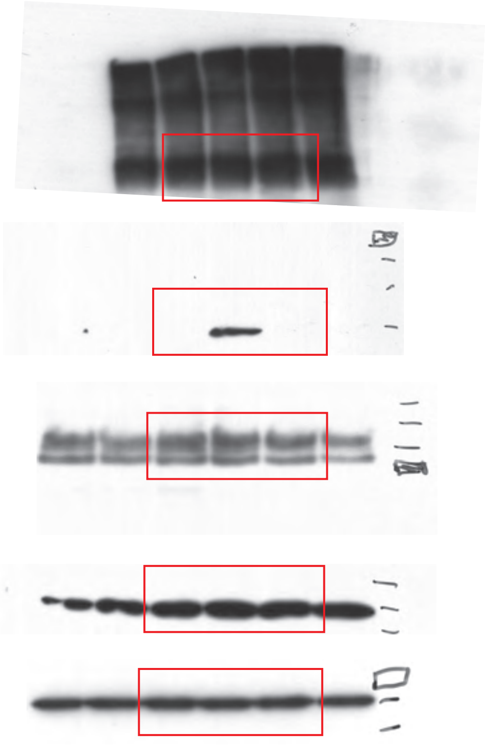

Supplementary Figure 8. Uncropped blots for figures 5a,6a,6b,6d and 7a. The cropped regions are denoted by red boxes.
